# Retinal Vessel Diameter Reductions Are Associated with Retinal Ganglion Cell Dysfunction, Thinning of the Ganglion Cell and Inner Plexiform Layers, and Decreased Visual Field Global Indices in Glaucoma Suspects †

**DOI:** 10.3390/diagnostics15131700

**Published:** 2025-07-03

**Authors:** Andrew Tirsi, Nicholas Leung, Rohun Gupta, Sungmin Hong, Derek Orshan, Joby Tsai, Corey Ross Lacher, Isabella Tello, Samuel Potash, Timothy Foster, Rushil Kumbhani, Celso Tello

**Affiliations:** 1 Department of Ophthalmology, Manhattan Eye, Ear, and Throat Hospital, Northwell Health, New York, NY 10065, USA; isabellatello112@gmail.com (I.T.); ctello@northwell.edu (C.T.); 2 Donald and Barbara Zucker School of Medicine at Hofstra/Northwell, Hempstead, NY 11549, USA; nleung1@northwell.edu (N.L.); rohunramgupta@gmail.com (R.G.); shong8@pride.hofstra.edu (S.H.); tfoster5@northwell.edu (T.F.); rkumbhani1@pride.hofstra.edu (R.K.); 3 Department of Ophthalmology, Larkin Community Hospital, Miami, FL 33431, USA; derekorshan@gmail.com; 4 Department of Ophthalmology, Broward Health, Deerfield Beach, FL 33064, USA; jtsai@browardhealth.org; 5 Department of Ophthalmology, Rutgers New Jersey Medical School, Newark, NJ 07103, USA; cl1193@njms.rutgers.edu; 6 Department of Ophthalmology, Rush University Medical Center, Chicago, IL 60612, USA; samuel.potash@einsteinmed.edu

**Keywords:** glaucoma, retinal vessel diameter, PERG, retinal ganglion cell, OCTA, Humphrey field analyzer global indices

## Abstract

**Background/Objectives**: The aim of this study was to evaluate the associations between optical coherence tomography angiography (OCTA)-based retinal vessel diameter (RVD) measurements, with retinal ganglion cell (RGC) function assessed by means of steady-state pattern electroretinography (ssPERG) using ganglion cell layer-inner plexiform layer thickness (GCL-IPLT) measurements and with Humphrey field analyzer (HFA) global indices in glaucoma suspects (GSs). **Methods**: Thirty-one eyes (20 participants) underwent a comprehensive ophthalmologic examination, ssPERG measurements utilizing the PERGLA paradigm, HFA, optical coherence tomography (OCT), and OCTA. The OCTA scans were processed using ImageJ software, Version 1.53, allowing for measurement of the RVD. Multiple linear regression models were used. **Results**: After controlling for age, race, central corneal thickness (CCT), and spherical equivalent (SE), a linear regression analysis found that the RVD explained the 4.7% variance in magnitude (Mag) (*p* = 0.169), 9.2% variance in magnitudeD (MagD) (*p* = 0.021), and 16.9% variance in magnitudeD/magnitude (*p* = 0.009). After controlling for age, CCT, and signal strength (SS), a linear regression analysis found that the RVD was significantly associated with the GCL-IPLT measurements (average GCL-IPL, minimum GCL-IPL, superior, superonasal, inferior, and inferonasal sectors) (*p* ≤ 0.023). An identical regression analysis where the RVD was replaced with the PERG parameters showed a significant association between the MagD and almost all GCI-IPLT measurements. RVD measurements were significantly associated with HFA VFI 24-2 (*p* = 0.004), MD 24-2 (*p* < 0.001), and PSD 24-2 (*p* = 0.009). **Conclusions**: Decreased RVD measurements were significantly associated with RGC dysfunction, decreased GCL-IPLT, and all HFA global indices in the GSs.

## 1. Introduction

Glaucoma, a leading cause of irreversible blindness worldwide, is a chronic, progressive optic neuropathy due to progressive loss of retinal ganglion cells (RGCs) [1,2,3]. Risk factors for glaucoma include but are not limited to older age, family history of glaucoma, being of the African American race, myopia, a thinner central corneal thickness (CCT), elevated intraocular pressure (IOP), and retinal vascular abnormalities [4]. Historically, the mechanical and vascular theories for glaucoma pathogenesis have predominated in the literature [5]. The mechanical theory proposes that glaucoma is mainly caused by increased IOP, leading to compression and damage to the lamina cribrosa and retinal axons, changes in the optic nerve head morphology, and visual field deficits [6]. However, IOP has been found to be lower than 22 mmHg in between 25 and 50% of patients diagnosed with primary open-angle glaucoma (POAG), and some patients face continued visual field deterioration despite adequate IOP control [4,7,8].

The vascular theory proposes that glaucoma is caused by insufficient ocular blood flow (OBF), vascular dysregulation, and vasospasm, leading to RGC dysfunction and cell death [9,10]. These chronic microvascular changes are hypothesized to dysregulate the balance between supply and demand for blood and oxygen delivery to RGCs. Abnormalities in retinal vasculature can result from decreased supply, as seen in structural vascular damage in hypertension, atherosclerosis, or inflammatory states. Alternatively, retinal vascular abnormalities can result from decreased demand secondary to dysfunctional RGCs that require less oxygen and blood. Both processes are hypothesized to affect the pathogenesis of glaucoma [11].

The retinal vessel diameter (RVD) can be measured via the central retinal artery equivalent (CRAE), central retinal vein equivalent (CRVE), and arteriolar-to-venous ratio (AVR). Earlier studies used fundus photographs to assess the RVD and used semi-automated computed software to measure arteriolar and venular calibers and calculate the AVR [12,13]. Retinal blood flow is controlled by various mediators for vasodilation and vasoconstriction of arterioles and venules, and it has been proposed that in systemic diseases, the retinal arterial caliber is more strongly influenced by hypertension, while retinal venular caliber is more strongly influenced by diabetes [14]. A decreased AVR has been found to be significantly associated with individuals with insulin resistance, [15] cerebral atrophy, [15] and hypertension [12].

Prior studies have investigated the association between RVD and glaucoma. Focal narrowing of retinal arterioles has been significantly associated with glaucoma and non-glaucomatous ischemic optic neuropathy [16]. Among POAG patients, retinal diameter narrowing has been associated with a decreased area of the neuroretinal rim, decreased retinal nerve fiber layer thickness, and visual field deficits [17]. The Blue Mountains Eye Study found that eyes with glaucomatous damage were at least two times more likely to have generalized arteriolar narrowing than eyes without glaucoma [18]. Adiarti et al. found that retinal arteriolar narrowing is associated with glaucomatous optic discs in young patients, independent of IOP increases [19]. While research has assessed the relationship between RVD changes and glaucoma, no study has yet examined the relationship between RVD changes and RGC function among asymptomatic patients at risk for developing glaucoma (glaucoma suspects (GSs)).

In recent years, optical coherence tomography-angiography (OCTA), a noninvasive, dye-free high-resolution imaging method, has provided objective assessments of the retinal microvasculature in the macula and in the parapapillary region, as well as in the choriocapillaris [20]. Li et al. found that reduced OCTA-measured peripapillary and macular vessel densities (VDs) were significantly lower in glaucoma and had statistically significant correlations with higher glaucoma stages [21]. Further literature shows OCTA-derived VDs to be significantly lower among eyes with mild-to-moderate POAG [22,23]. While OCTA-derived VDs have been investigated, OCTA-measured RVDs have not been studied in the GS population.

Reliable and accurate monitoring of RGC function is helpful when investigating the vascular contributions to glaucoma. Studies have shown that steady-state pattern electroretinography (ssPERG) allows for noninvasive monitoring of RGC function [24]. Porciatti and Ventura created the PERGLA paradigm, allowing for a patient-centered and user-friendly method of ssPERG to use for clinical detection of glaucoma with similar reliability to PERG metrics gained from protocols utilizing corneal electrodes [25]. Gillmann et al. examined the repeatability and reproducibility of the PERGLA paradigm and found that both ssPERG parameters (magnitude and magnitudeD) were significantly repeatable and reproducible [26]. Utilizing the PERGLA protocol, Banitt et al. found that ssPERG has been able to detect RGC dysfunction 8 years before changes in the retinal nerve fiber layer (RNFL) [24]. Ventura et al. found a significant change in ssPERG phase in GSs while the amplitude remained within normal limits [27]. Gordon et al. demonstrated that the age-corrected PERG amplitude decreases in both untreated and treated GSs over 10 years on average [28]. Mavilio et al. demonstrated a significant reduction in ssPERG amplitude and phase in GSs and early glaucoma patients relative to normal patients [29].

The purpose of this prospective, observational cross-sectional study was to examine the relationship between RGC function and the RVD, assessed by means of ssPERG and OCTA, respectively, in the GS population. Additionally, the relationships among SD-OCT thickness measurements, Humphrey visual field analyzer (HFA) global indices, the RVD, and ssPERG parameters were evaluated.

## 2. Methods

### 2.1. Participants

In this observational cross-sectional study, 20 glaucoma suspects (31 eyes) were recruited from the larger ongoing longitudinal GS study assessing the risk of progression of glaucoma by means of ssPERG (IRB #18-0397), conducted by the Department of Ophthalmology at the Manhattan Eye Ear and Throat Hospital. The institutional review boards of Northwell Health approved the protocol, and this methodology adhered to the tenets of the Declaration of Helsinki for research involving human subjects and the Health Insurance Portability and Accountability Act. Written informed consent was obtained from every participant.

All patients underwent a complete ophthalmological examination including slit lamp biomicroscopy, fundoscopy, IOP measurements via Goldmann applanation tonometry, and central corneal thickness (CCT) measurements with ultrasound pachymetry (Accutome Inc., PachPen, Malverne, PA, USA).

A diagnosis of suspected glaucoma was established as per the American Academy of Ophthalmology’s Preferred Practice Guidelines for POAG suspects [30]. Subjects with the presence of consistently elevated IOP or a suspicious optic nerve, retinal nerve fiber layer (RNFL), or visual field in one or both eyes were included in the study. Risk factors for POAG include older age, being of the African race or Latino or Hispanic ethnicity, elevated IOP, family history of glaucoma, lower ocular perfusion pressure, type 2 diabetes mellitus, and thin central corneas.

Using the Humphrey field analyzer (HFA) 24-2 SITA-standard test, only participants at stage 0 (no VF losses) based on the Glaucoma Staging System (GSS 2) were enrolled in this study [31]. A normal HFA test was defined by a Glaucoma Hemifield Test (GHT) “within normal limits”, with a pattern standard deviation (PSD) within 95% confidence limits and mean deviation (MD) ≥ −2 dB. Participants with unreliable HFA global indices, fixation losses, or a false positive rate or false negative rate > 20% were excluded. Participants with prior intraocular surgeries, except uncomplicated cataract extraction, ocular trauma, or ocular or systemic conditions that may have affected the ONH or retinal structural morphology or function, were excluded. No participants received IOP lowering treatment at the time of enrollment. OCT images with low quality, visible eye motion, blinking artifacts, or algorithm segmentation failures were not included in the study. One eye was diagnosed with an epiretinal membrane, three eyes were removed for low quality OCT-A images, and five eyes were removed because of abnormal visual field testing (early glaucoma). As a result, nine eyes in total were excluded from the study. No preperimetric glaucoma subjects were enrolled.

The exclusion criteria for this study included the following: pupil diameter <3 mm, large refractive errors > ±5.0 diopters, and cylinders < ±3.0 diopters. The demographic characteristics for the study subjects are included in Table 1.

### 2.2. OCT Measurements

Both the ganglion cell layer-inner plexiform layer (GCL-IPL) and RNFL images were obtained using the macular 200 × 200 cube and optic disc 200 × 200 cube scans, respectively, with the Cirrus HD-OCT device. Explicit details of the HD-OCT scan protocols have been described in detail previously [26]. The RNFL algorithm within the Cirrus HD-OCT was used to measure the peripapillary RNFL thicknesses (both averages and quadrants) automatically [32]. The ganglion cell analysis (GCA) algorithm utilized in the HD-OCT device processes and measures the thickness of macular GCL-IPL as an average and minimum and in 6 sectors (superotemporal, superior, superonasal, inferonasal, inferior, and inferotemporal). The GCA algorithm has been previously described [33]. These were measured from the elliptical annulus centered on the fovea. The only images with a signal strength >7 in both the macular and optic disc cube scans were included in this study. Images with motion artifacts, poor centering, or segmentation errors were excluded from the study.

### 2.3. Optical Coherence Tomography Angiography (OCTA) Measurements of Vessel Diameter

The macular 3 × 3 mm OCTA scans were collected via a Zeiss Cirrus HD OCTA 5000 AngioPlex device (Carl Zeiss Meditec, Inc., Dublin, CA 94568, USA). The AngioPlex utilizes an OCT-microangiography complex algorithm (OMAG) and an A-scan rate of 68 Khz [34].

The open-source package ImageJ (version 1.53) was used to calculate the average retinal vessel diameter in a selected region (in mm). Vessel Analysis is a plugin which automatically calculates vascular diameter metrics, which is the ratio of the skeletonized vasculature area to the total area, according to the following formula: vascular length density measurements = skeletonized vessel area/total area × 100% [35].

**Figure 1:**
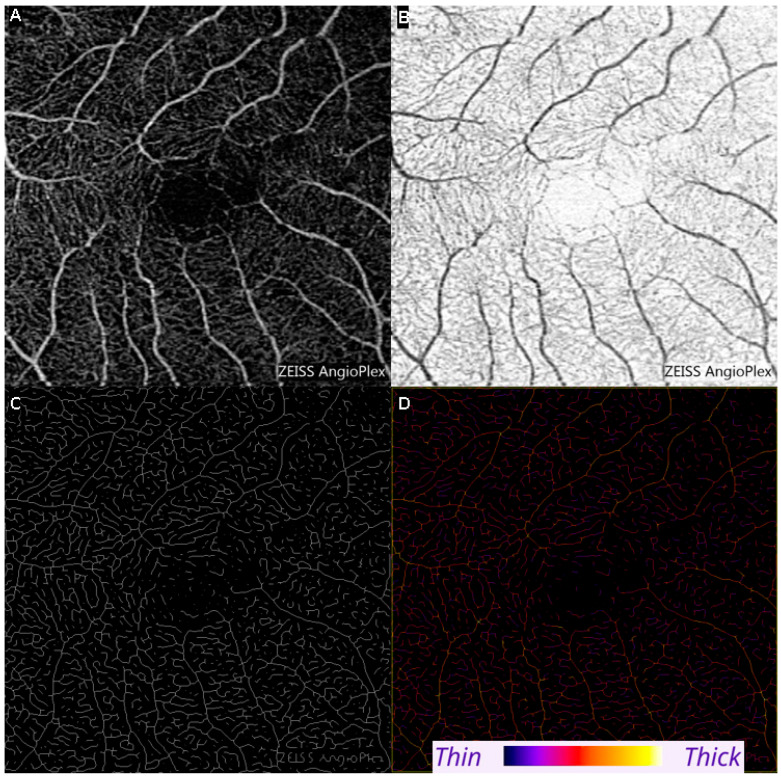
The methodology of how the Vessel Analysis software on ImageJ calculates retinal vessel diameter. Top left (**A**) is the unfiltered image of the superficial plexus of the retinal vasculature. The image is binarized (**B**) and subsequently skeletonized (**C**) The Image Analysis software converts the black and white skeletonized image into a colorized skeletonized image (**D**). The color scale represents different degrees of vessel thickness.

**Figure 2:**
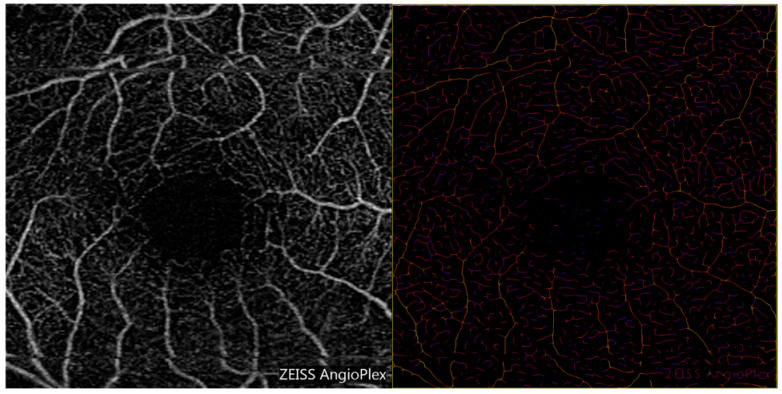
The left image is the unfiltered OCTA image of the superficial plexus. The right image is the same image processed by the Vessel Analysis software for ImageJ. This subject had lower retinal vessel diameter relative to the other glaucoma suspects.

**Figure 3:**
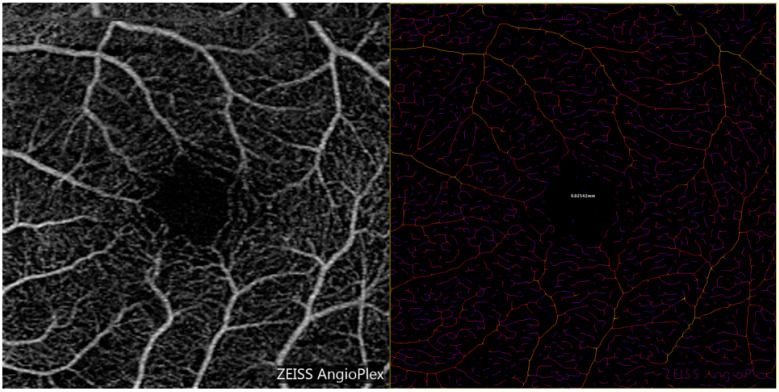
The left image is the unfiltered OCTA image of the superficial plexus. The right image is the same image processed by the Vessel Analysis software for ImageJ. This subject had higher retinal vessel diameter relative to the other glaucoma suspects.

Each OCTA image was inverted in color per the instructions of the Vessel Analysis user manual [35,36]. A region of interest was selected, and the software generated a colored, skeletonized image (see Figure 1, Figure 2 and Figure 3). Each pixel’s color represents the relative thickness of the vessel in that area. Thicker vessels range from white to orange in color, whereas thinner vessels vary from red to purple (please see image 3 and the color scale for reference). After inputting measurements for scaling, the “distance in mm” was set to 3, and the “distance in pixels” was set to 1024. The corresponding pixel width of the image was calculated by Fiji. To avoid errors due to image magnification, the images were analyzed after rescaling the pixel ratio to the millimeter scale using the known distance of the fovea to the optic disc in the set scale option of the software. Specifically, the dimensions of the image were set to 1024 pixels by 1024 pixels, with 341.33 pixels representing one millimeter [35]. This data was used to formulate RVD measurements for each eye.

### 2.4. Steady State Pattern Electroretinography

Diopsys NOVA ssPERG (Diopsys, Cedar Knolls, NJ, USA) was used in accordance with the PERGLA paradigm outlined by Porciatti and Ventura [25]. The use of ssPERG has been shown to have high test-retest repeatability [37]. The PERGLA protocol adds filters and amplifiers to ssPERG recordings to achieve an amplitude and signal-to-noise ratio adherent to the International Society for Clinical Electrophysiology of Vision (ISCEV) standards [38].

The ssPERG was recorded using a commercially available system—Diopsys^®^ NOVA-PERG (Diopsys, Inc., Cedar Knolls, NJ, USA)—as described previously [39,40,41,42,43].

Three PERG measurements (Mag, MagD, and MagD/Mag ratio) were displayed as test results. The Mag (mV) represents the amplitude of the signal strength at the specific reversal rate of 15 Hz in the frequency domain, while MagD (mV) represents an adjusted amplitude of the signal for the phase variability throughout the waveform recording [42]. The MagD/Mag ratio is a ratio that is a within-subject representation of the phase consistency of ssPERG [42]. The MagD/Mag ratio also demonstrates how the phase changes are affecting the Mag.

Recent research has demonstrated that these ssPERG parameters are repeatable, reproducible, and sufficiently reliable in clinical practice [26]. The results were also presented in a color-coded system similar to a “traffic light system”, with green showing the results within the reference range, yellow representing values within the borderline reference range, and red representing results outside the reference range. Please see the table below for the normal, borderline, and abnormal ssPERG values based on Diopsys normative data. The PERG device and software used for this investigation were provided by Diopsys Inc., Middletown, PA, USA.
**Results****Mag****MagD****MagD/Mag ratio**Within reference range>1.0>0.752>0.752Borderline reference range0.7–1.00.45–0.7520.642–0.752Abnormal <0.7<0.45<0.642Mag—Magnitude, MagD—MagnitudeD, MagD/Mag ratio—MagnitudeD over Magnitude ratio.

### 2.5. Statistical Analysis

For all ssPERG and RVD variables, outliers with values ≥3 standard deviations from the mean were excluded from the analyses. The Shapiro–Wilk test was used to determine the normality of the distribution for all important variables. Descriptive statistics were used to evaluate continuous and demographic data. The mean and standard deviation values were determined for each ssPERG parameter (Mag, MagD, and MagD/Mag ratio), HFA SITA standard (24-2) tests, and all RNFLT variables. Chi-squared tests were used for categorical variables. We calculated the relationships between the RVD and GCL-IPL measurements with a two-step linear regression analysis. All values are presented as mean values ±95% confidence interval. For all analyses, a *p* value < 0.05 was considered statistically significant.

In the linear regression analysis examining the relationship between the RVD and the ssPERG parameters, age, spherical equivalent (SE), race, and CCT were entered as covariates (co-founding factors) in the first step of the model, and individual ssPERG parameters (Mag, MagD, and MagD/Mag ratio) were entered in the second step. Ventura et al. reported that an older age was strongly associated with a reduced PERG amplitude [44]. Oner et al. reported a reduction in PERG amplitude in myopic eyes, with this effect increasing as the ocular axial length increased [45]. Ventura et al. reported that the African American race was associated with lower PERG amplitudes in GSs [44]. The central corneal thickness was controlled due to its significance as a risk factor for glaucoma [46].

In examining the relationship between the GCL-IPL thickness measurements and the RVD and ssPERG, we controlled for age, signal strength, and CCT. Ooto et al. demonstrated that RNFL, GCL, and IPL thicknesses decrease with increasing age [47]. Decreased signal strength has been associated with underestimation of RNFL and macular thickness measurements, with the OCT scan quality being the strongest factor seen with interscan variability in tissue thickness measurements [48]. In an identical linear regression model, while examining the relationship between HFA global indices and RVD, we controlled for age, CCT, and SE in step 1, and the RVD was entered in step 2.

The statistical analysis was performed using SPSS software version 28.0 (SPSS Inc., Chicago, IL, USA).

## 3. Results

In this study, a total of 31 eyes from 20 glaucoma suspect patients met the initial inclusion criteria. Table 1 and Table 2 contain the demographics and structural and functional parameter characteristics of the study subjects. Measurements of the average RNFL and average GCL+IPL thicknesses were found to be in line with previous studies (RNFL thickness = 89.84 +/− 9.54 µm, GCL+IPL thickness = 78.57 +/− 6.74 µm). 

### 3.1. Relationship Between Retinal Vessel Diameter and ssPERG Parameters

**Figure 4:**
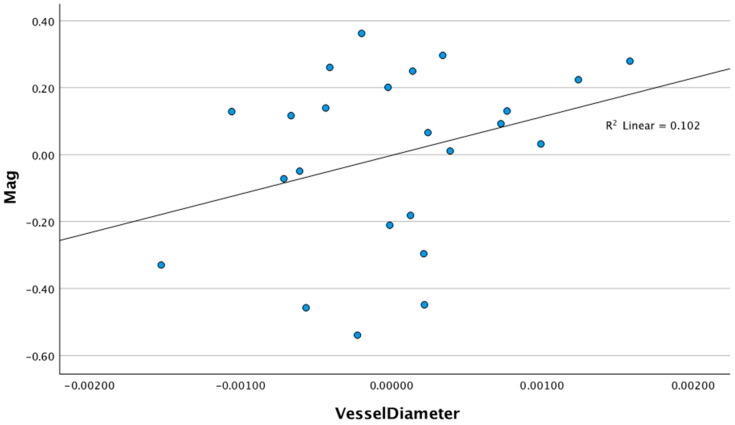
Scatter plot of the relationship between magnitude and retinal vessel diameter after adjusting for age, SE, race, and CCT (r^2^ = 0.102, *p* = 0.169).

**Figure 5:**
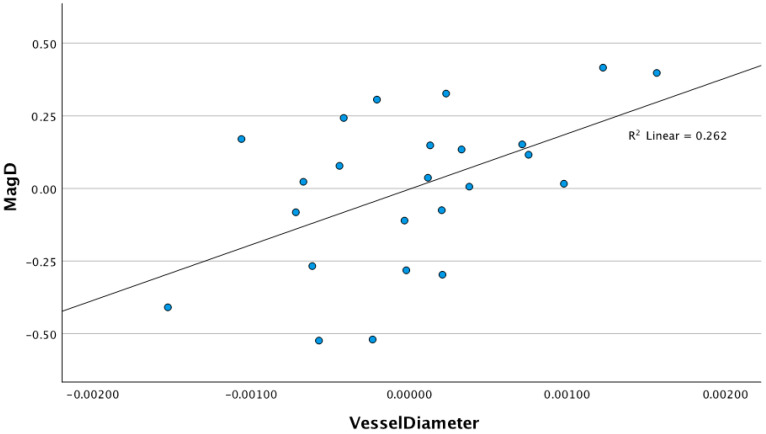
Scatter plot of the relationship between magnitudeD and retinal vessel diameter after adjusting for age, SE, race, and CCT (r^2^ = 0.262, *p* = 0.021).

**Figure 6:**
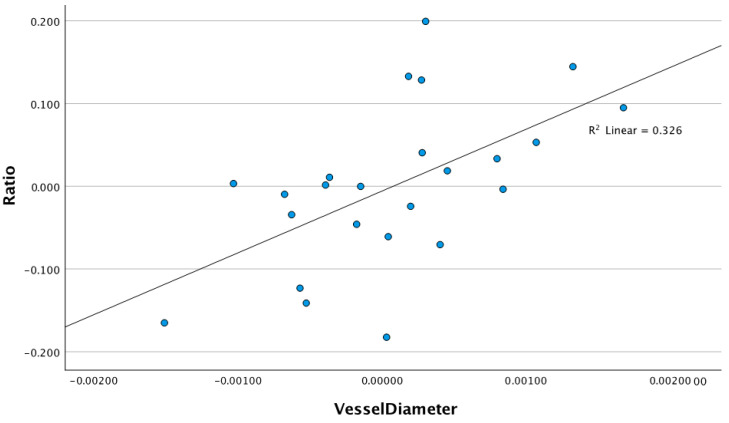
Scatter plot fo the relationship between magnitudeD/magnitude ratio and retinal vessel diameter after adjusting for age, SE, race, and CCT (r^2^ = 0.322, *p* = 0.009).

In three separate linear regression models controlling for age, race, SE, and CCT (step 1), the RVD (step 2) explained an additional and significant 9.2% variance in the MagD (B = 190.34 (CI 95%: 31.95–348.74), *p* = 0.021) and 16.9% variance in the MagD/Mag ratio (B = 76.55 (CI 95%: 22.01–131.08) *p* = 0.009). The retinal vessel diameter did not explain any significant variance in the Mag (B = 115.50 (CI 95%: −53.95–284.96) *p* = 0.169) (Table 3). The linear regression models examining the relationship between the RVD and the Mag (R^2^ linear = 0.102), MagD (R^2^ Linear = 0.262), and MagD/Mag ratio (R^2^ Linear = 0.326) are shown in the scatter plots in Figure 4, Figure 5 and Figure 6, respectively.

### 3.2. Relationship Between Retinal Vessel Diameter and Ganglion Cell Layer-Inner Plexiform Layer Measurements

Utilizing a two-step linear regression model where the GCL-ILP thickness measurements were entered as dependent variables one after another, and after controlling for age, signal strength, CCT (step 1), and RVD (step 2), 28.6% variance in the average GCL-IPL *(B = 4039.4 (CI 95: 1291.2–6787.6), p = 0.006), 37.1%* variance in the minimum GCL-IPL *(B = 4759.9 (CI 95%: 1966.6–7553.1), p = 0.002), 23.1%* variance in the superior sector GCL-IPL *(B = 3792.0 (CI 95%: 1139.0–6445.0), p = 0.007), 20.6%* variance in the superonasal sector GCL-IPL *(B = 3865.8 (CI 95%: 859.5–6872.2), p = 0.014)*, *16.4%* variance in the inferior sector GCL-IPL *(B = 3286.4.7 (CI 95% 497.4–6075.5), p = 0.033)*, and *19.6%* variance in the inferonasal sector GCL-IPL *(B = 3810.6 (872.3–6748.9), p = 0.014)* were significantly explained (Table 4). In the same regression analysis, the RVD did not significantly explain the variance in two GCL-IPL sectors: the inferotemporal sector GCL-IPL variance of *8.4% (B = 2225.5 (CI 95%: −142.9–4593.9), p = 0.064)* and the superotemporal sector GCL-IPL variance of *11.0% (B = 2466.2 (CI 95%: −346.2–5278.8), p = 0.082)* (Table 4).

### 3.3. Relationship Between Retinal Vessel Diameter and Humphrey Field Analyzer Global Indices

Using a two-step linear regression model with HFA global indices as the dependent variables and controlling for age, CCT, spherical equivalent (SE) (step 1), and RVD (step 2) significantly explained the 34.7% variance in HVF VFI 24-2 (B = 743.2 (CI 95%: 273.8–1212.6), *p* = 0.004), 57.2% variance in HVF MD 24-2 (B = 975.5 (CI 95%: 576.2–1374.9), *p* < 0.001), and 26.5% variance in HVF PSD 24-2 (B = −235.3 (CI 95%: −404.5–−66.2), *p* = 0.009). The central corneal thickness was controlled for, because thinner corneas have been shown to be significantly associated with increased loss in MD and PSD [49]. The spherical equivalent was controlled for, because myopia has been shown to affect HVF measurements [49].

### 3.4. Relationship Between ssPERG Parameters and Ganglion Cell Layer-Inner Plexiform Layer Thickness Measurements (Table 5)

In an identical two-step linear regression model, where GCL-ILP thickness measurements were entered as dependent variables, and after controlling for age, signal strength, CCT (step 1), and Mag (step 2), 16.5% variance in the superior sector GCL-IPL *(B = 7.9 (CI 95%: 2.7–13.1) p = 0.005), 7.6%* variance in the superonasal sector GCL-IPL *(B = 5.8 (CI 95%: 0.1–11.5), p = 0.047), 17.0%* variance in the superotemporal sector GCL-IPL *(B = 7.6 (CI 95%: 2.0–13.1), p = 0.01)*, and *8.4%* variance in the inferonasal sector GCL-IPL *(B = 6.1 (CI 95%: 1.1–11.2), p = 0.02)* were significantly explained. The magnitude did not significantly explain any variance in the average, minimum, inferior and inferotemoral thickness measurements (Table 5).

**Table 5:** Associations of ganglion cell layer-inner plexiform layer thickness measurements with magnitude, controlling for age, signal strength, and central corneal thickness.

	Step 1 (Age, Signal Strength, and CCT)		Step 2 (Magnitude)			
GCL-IPLT Sectors	ΔR^2^	B (95% CI)	ΔR^2^	B (95% CI)	R^2^	SE
Average	0.521 **	112.5 (71.5–153.4)	0.071	4.9 (−0.8–10.7)	0.521	4.841
Minimum	0.445 **	97.5 (51.9–143.2)	0.093	5.9 (−0.4–12.2)	0.538	5.050
Superior	0.522 **	125.2 (82.4–167.9)	0.165 **	7.9 (2.7–13.1)	0.687	4.197
Superonasal	0.605 **	125.1 (83.2–167.0)	0.076 *	5.8 (0.1–11.5)	0.681	4.574
Superotemporal	0.432 **	108.6 (64.6–152.6)	0.170 **	7.6 (2.0–13.1)	0.601	4.472
Inferior	0.801 **	134.9 (106.6–163.2)	0.013	2.3 (−1.9–6.4)	0.814	3.324
Inferonasal	0.668 **	131.4 (92.6–170.3)	0.084 *	6.1 (1.1–11.2)	0.752	4.078
Inferotemporal	0.669 **	105.6 (74.3–136.8)	0.020	2.4 (−2.1–7.0)	0.689	3.670

CCT = central corneal thickness, GCL-IPLT = ganglion cell layer-inner plexiform layer thickness. Steps of the regression analysis: ΔR^2^ is the change in R^2^, B (95% CI) is the B coefficient and 95% confidence interval ranges, R^2^ is the total R2 of the model, and SE is the standard error of the estimates of the final model. * *p* < 0.05. ** *p* < 0.01.

After replacing the Mag with the MagD as a predictor in an identical regression model, and after controlling for age, signal strength, CCT (step 1), and MagD (step 2) *13.0%* variance for the average GCL-IPL *(B = 5.9 (CI 95%: 1.3–10.6), p = 0.015), 18.5%* variance for the minimum GCL-IPL *(B = 7.3 (CI 95%: 2.3–12.3), p = 0.006), 17.9%* variance in the superior sector GCL-IPL *(B = 7.3 (CI 95%: 2.8–11.8), p = 0.003), 17.3%* variance in the superotemporal sector GCL-IPL *(B = 6.8 (CI 95%: 1.9–11.7), p = 0.009)*, and *6.2%* variance in the inferonasal sector *(B = 4.7 (CI 95%: 0.0–9.4), p = 0.050)* were explained (Table 6).

In a third model using the MagD/Mag ratio (Step 2) as a predictor, the MagD/Mag ratio significantly explained the *18.5%* variance in average GCL-IPL *(B = 24.6 (CI 95%: 9.7–39.5), p = 0.003), 29.8%* variance in minimum GCL-IPL *(B = 32.4 (CI 95%: 17.9–46.8), p < 0.001)*, and *9.3%* variance in the superior sector GCL-IPL *(B = 18.2 (CI 95%: 0.4–36.0), p = 0.046)* (Table 7). Insignificant relationships utilizing the same two-step linear regression model demonstrated *7.5%* variance in the superotemporal sector GCL-IPL *(B = 15.5 (CI 95%: −3.5–34.6), p = 0.104), 1.7%* variance in the superonasal sector GCL-IPL *(B = 8.4 (CI 95%: −10.7–27.4), p = 0.369), 1.3%* variance in the inferior sector GCL-IPL *(B = 7.1 (CI 95%: −5.6–19.8), p = 0.257), 1.4%* variance in the inferonasal sector GCL-IPL *(B = 7.7 (CI 95%: −9.9–24.9), p = 0.371)*, and *4.8%* variance in the inferotemporal sector GCL-IPL *(B = 11.5 (CI 95%: −1.9–24.9), p = 0.088)*.

## 4. Discussion

In this study, we report that reductions in retinal vessel diameters were significantly associated with a reduction in ssPERG parameters (magnitudeD and magnitudeD/magnitude ratio) in GSs, demonstrating the presence of a vascular dysregulation associated with RGC dysfunction. Furthermore, we found that a decrease in RVD was significantly associated with a decrease in most sectoral GCL-IPL thickness measurements, suggesting either an ischemic basis for glaucomatous structural damage or the presence of secondary vascular constriction due to decreased demands from dysfunctional or dead RGCs. We also report a significant association between the RVD and all HFA global indices, suggesting that vascular dysregulation is associated with a significant change in visual field test indices (VFI24-2, MD24-2, and PSD24-2). Finally, we report that decreased ssPERG parameters were significantly associated with decreases in the sectoral GCL-IPL thickness measurements in GSs. This relationship between the average GCL-IPL thickness and ssPERG parameters was significant, despite the average GCL-IPL being within the normal range of values [33,39].

This study’s findings add to the existing literature stating that retinal vessel diameters change in POAG, and this is mostly driven by a reduction in retinal arterioles rather than venules. The Beijing Eye Study showed significantly narrower retinal arteries but no significant difference in retinal vein diameters [50]. Adiarti et al. found that the mean CRAE was significantly narrower in patients with glaucomatous optic discs compared with healthy patients, and no significant differences in CRVE were found between the two groups [19]. Lee et al. found that in patients with bilateral normal-tension glaucoma with asymmetric progression, narrowing of the retinal arteriolar caliber was significantly associated with glaucoma-progressed eyes, but no correlation was found for the retinal venular caliber [51]. In this study, we used ImageJ software to measure the RVD from OCTA macular scans, with the majority of the contributions to RVD measurements coming from retinal arteriolar diameters. These findings suggest that a significant change in retinal vessel diameters is associated with RGC dysfunction, reflecting either an ischemic basis for RGC dysfunction or a possible secondary arteriolar vasoconstriction due to fewer viable RGCs and therefore fewer RGCs to nourish [52].

Retinal blood flow is tightly autoregulated to meet the metabolic demands of RGCs. One proposed biological mechanism for decreased retinal arteriolar caliber in glaucoma is the theory of supply vs. demand for blood flow to RGCs [53]. On the one hand, RGC dysfunction can lead to a decreased metabolic need for oxygen and other nutrients, causing decreased blood flow and vasoconstriction of vessels. Alternatively, the underlying cause of RGC dysfunction can be attributable to pathology of the retinal vessels themselves due to impaired autoregulation, vasoactive substance leakage, and vasoconstriction [54,55,56]. Glaucoma has been associated with endothelial dysfunction and decreased levels of nitric oxide, endothelin-1 (ET-1), vascular endothelial growth factor (VEGF), and soluble VEGF receptor FLT-1 [57,58]. Emre et al. found an association of increased plasma endothelin-1 levels in patients with progressive POAG versus stable POAG [59]. Both endothelin-1 and nitric oxide stimulate angiogenesis and consequently promote vascular tortuosity [60]. Decreased levels of these factors are thus associated with decreased tortuosity, which Wu et al. demonstrated was associated with glaucoma as straighter retinal vessels had increased thinning of the neuroretinal rim [61].

We believe that the decreases in the RVD demonstrated in this study were likely due to a combination of primary and secondary endothelial dysfunction that limits perfusion of the superficial capillary plexus. Chronic ischemia to inner retinal layers and to RGCs would subsequently lead to RGC dysfunction and their morphological changes, including decreased soma size, trimming of the dendritic tree, and reduced retinal nerve fiber diameters preceding cell death [39]. This is further reflected in our finding that the RVD was significantly associated with decreased average and sectorial GCL-IPL thickness measurements, which are evidence of RGC morphological changes.

While the Diopsys user manual suggests that the MagD/Mag ratio is the most sensitive marker of glaucoma, Gillmann et al. examined the PERGLA paradigm and reported that the MagD/Mag ratio has decreased reliability and repeatability relative to Mag and MagD in the RGCs of healthy subjects [26]. The PERGLA paradigm further demonstrated that the amplitude (Mag) and phase (MagD) are uncoupled, indicating that the amplitude and phase exhibit different aspects of neuronal activity [62]. Porciatti and Ventura stated that a faster steady-state stimulus over a slower transient stimulus can demonstrate glaucomatous dysfunction due to its subjugation of RGCs to greater metabolic stress, allowing for uncoupling of the amplitude and phase [25].

### 4.1. Retinal Vessel Diameters and Their Associations with ssPERG Parameters

Retinal ganglion cell dysfunction would be expected to occur before programmed cell death, also called apoptosis. RGC apoptosis occurs due to a cellular insult (inflammation, ischemia, etc.) followed by programmed cell death [63]. RGC degeneration presents a similar pattern to central nervous system neuronal cell dysfunction, with a reduction in retrograde axonal transport of neurotrophic factors from the brain to the RGCs initiating the degeneration process [64]. Alteration of functional activity, including reduction in light sensitivity and reduction of dendritic arbor excitatory synapses, will occur [65]. We theorize that the RGC dysfunction found here in the GS population by means of ssPERG was partially due to a reduction in blood supply perfusing the retinal ganglion cell layer of the inner retina. A second theory would be that the retinal arteriolar constriction could be secondary to the fact that stressed and dysfunctional RGCs would demand less blood for metabolism, and therefore an arteriolar constriction would follow.

Hagen–Poiseuille’s equation on blood flow states that resistance is inversely related to the cross-sectional area of vasculature and inversely related to the fourth power of the vessel radius. Therefore, small decreases in the vessel diameter led to substantial increases in vascular resistance and thus a reduction in the delivery of oxygen and other necessary molecules. The flow dynamics within retinal circulation do not follow Poiseuille’s law exactly, as blood is a non-Newtonian fluid. Additionally, smaller arteriolar vessels are influenced by shear forces and physical interactions more than the laminar flow seen in larger vessels [66]. This smaller vessel diameter causes the velocity near the vessel wall to be nearly zero and the velocity distribution across the vessel to be parabolic [66]. Even after adjusting for these variables of retinal circulation, the Hagen–Poiseuille equation still shows significant changes in flow with small changes in the vessel diameter [67].

In this study, we report a significant association between the RVD and MagD and subsequently the MagD/Mag ratio, suggesting that reduced blood flow to RGCs and chronic ischemia trigger RGC dysfunction in the form of increased latency only. This is clinically significant because it indicates that Mag, the ssPERG parameter marker for RGC death, was not yet associated with the RVD, due the population of this study being GSs with normal HFA indices and normal RNFL thickness measurements and the glaucomatous disease being at its earliest stages with presumably no signs of RGC loss.

### 4.2. Retinal Vessel Diameter and Its Association with Sectoral GCL-IPL Thickness Measurements and HVF 24-2 Global Indices

We found that a decrease in the retinal vessel diameters was significantly associated with a decrease in almost all GCL-IPL thickness parameters, after controlling for age and other covariates. These findings correspond with Tham’s findings of sparser retinal vasculature associated with thinner GCL-IPL thicknesses [60]. Within the GS population, Shin et al. detected significant GCL-IPL progressive thinning in the GS population during a 5.7-year follow-up [68]. The progressive GCL-IPL thinning was detected significantly earlier than the development of visual field defects in this population [68].

In this study, we found a strong association between the retinal vessel diameter and HFA global indices VFI24-2, MD24-2, and PSD24-2. Our findings are in line with those of Hall et al. and De Leon et al., who found associations between the retinal arteriolar diameter and localized visual field defects in POAG patients [52,69]. Lam et al. found that the presence of peripapillary focal arteriolar narrowing was related to the severity of visual field loss in glaucoma patients [70]. These findings differ from those of Chiquet et al. and Rao et al., who found no significant relationship between the RVD and visual field indices; however, these studies examined POAG patients as opposed to GS patients [71,72].

### 4.3. Associations Between SSPERG Parameters and GCL-IPL Thickness Measurements

Decreases in the magnitude, magnitudeD, and magnitudeD/Magnitude ratio were significantly associated with decreases in the sectoral GCL-IPL thickness measurements in GSs. This relationship between the average GCL-IPL thickness and ssPERG parameters was significant despite the average GCL-IPL being within the normal range of values [33]. Zivkovic et al. found significant decreases in the GCL+IPL thickness in relation to glaucoma stages (early, moderate, or severe) [73]. To the best of our knowledge, this is the first study to examine the relationship between the RVD, GCL-IPL thickness measurements, and Humphrey Visual Field Analyzer 24-2 global indices in GSs.

Zhou et al. measured the RVD in POAG, and they concluded that chronic vasoconstriction limits the energy supply to the retinal and cerebral neurones, with susequent hypo-metabolism leading to neuronal cell death.The authors proposed that the root cause of POAG is primarily of a vascular origin [74]. Hwang et al. studied the relationships between IOP and blood flow and their effects on structural and functional damage [75]. They offered three possible scenarios. The first two scenarios highlight the possibility that either neural tissue loss drives reductions in blood flow through decreased metabolic demand or neural structural loss is a consequence of ischemic damage due to impaired perfusion. The thirst scenario describes decreased blood flow as an independent cause of visual field loss [75]. In this study, we believe the first two scenarios to be more applicable and that the RVD could lead to a chronic decrease in blood flow with susequent structural and functional losses. Cheng et al. found a direct relationship between decreased RVDs and structural damage, as most RVD changes were associated with changes in temporal sectors. In this study, we reported similar patterns of structural damage [76].

Furthermore, RVD measurements have similar diagnostic ability for detecting glaucoma to the average RNFL thickness, and they may help clinicians evaluate the risk of conversion in GSs [77,78].

We acknowledge certain limitations in this study. First, our sample size of GSs was small. Second, the cross-sectional nature of the study limits the ability to extrapolate how changes in the RVD relate to changes in ssPERG amplitude and phase over time. Third, the vessel analysis software in ImageJ is new and examines the RVD differently from the CRAE and CRVE methods previously utilized in the literature, preventing direct comparison to this study. However, this method of RVD measurement is an objective structural and functional test, as the whole unprocessed OCTA image is utilized by the Vessel Analysis software, generating only one measurement and giving this methodology a possible advantage over other methods. Vessel caliber measurements in the form of CRAE and CRVE require the users to choose specific vessels of interest and measure subjectively a localized area on the vessel. Fourth, the OCTA was conducted on non-dilated patients. No data on blood pressure, nicotine use, or caffeine intake was collected, which can affect the autoregulation of the retinal vasculature. Ocular pressure should be used in future studies to better understand these relationships.

## 5. Conclusions

This prospective, observational cross-sectional study demonstrated that glaucoma suspects with decreased RVDs were associated with RGC dysfunction, with the main mechanism being attributed to increased latency. Furthermore, RVD measurements and ssPERG parameters were associated with decreases in almost all GCL-IPL thickness measurements, demonstrating the relationship between impaired blood supply, RGC dysfunction, and subsequent morphological changes to RGCs found in the OCT thickness measurements. Further studies should examine the longitudinal relationship of the RVD, ssPERG, GCL-IPL, vessel density, ocular perfusion pressure, and HFA as patients convert from GSs to preperimetric glaucoma or POAG.

## Figures and Tables

**Table 1:** Demographics and ocular characteristics of study subjects (*n* = 31 eyes, 20 subjects).

	Mean	Minimum	Maximum
Age (years)	59.89 ± 14.20	28	78
Sex (% females)	20 F (64.51%)	N/A	N/A
Race C/H/A	25/4/2	N/A	N/A
IOP (mm Hg)	18.29 ± 4.46	10	26
CCT (µm)	551.67 ± 29.22	475	593
SE (diopters)	−0.53 ± 2.34	−5.75	4
Humphrey Field Analyzer global indices			
HFA MD 24-2 (dB)	0.32 ± 0.94	−2.86	1.60
HFA PSD 24-2 (dB)	1.52 ± 0.33	0.90	2.43
HFA VFI 24-2 (%)	99.25 ± 0.63	96	100
Steady state pattern electroretinography			
Magnitude (mv)	1.62 ± 0.54	0.97	3.45
MagnitudeD (mv)	1.34 ± 0.58	0.50	2.90
MagD/Mag ratio	0.81 ± 0.14	0.43	0.87
OCT-A based retinal vessel diameter			
Vessel diameter (µm)	2.42 ± 0.098	2.27	2.64

F = females, C = Caucasian, H = Hispanic, A = Asian, IOP = intraocular pressure, CCT = central corneal thickness, SE = spherical equivalent, HFA = Humphrey field analyzer, MD = mean deviation, PSD = pattern standard deviation, VFI = visual field index, Mag = magnitude, MagD = MagnitudeD, OCT-A = optical coherence tomography-angiography.

**Table 2:** Optical coherence tomography thickness measurements (µm) (*n* = 31 eyes, 20 subjects).

Quadrants or Sectors	Mean	Minimum	Maximum
RNFL thickness measurements by quadrants			
Average	89.84 ± 9.54	77	113
Superior quadrant	105.29 ± 16.39	73	79
Temporal quadrant	65.38 ± 11.56	50	102
Inferior quadrant	117.10 ± 13.50	88	146
Nasal quadrant	69.94 ± 8.50	57	88
Ganglion cell layer-inner plexiform layer thickness by sectors			
Average	78.57 ± 6.74	64	90
Minimum	76.74 ± 6.63	61	89
Inferior sector	79.41 ± 6.77	59	90
Inferonasal sector	78.67 ± 7.56	63	92
Inferotemporal sector	79.57 ± 6.54	65	91
Superior sector	77.64 ± 7.09	63	92
Superonasal sector	81.03 ± 10.13	64	92
Superotemporal sector	77.58 ± 7.39	64	94

**Table 3:** Associations of magnitude, magnitudeD, and magnitudeD/magnitude ratio with retinal vessel diameter measurements, controlling for age, race, central corneal thickness, and spherical equivalent.

	Step 1 (Age, Race, CCT, SE)		Step 2 (Vessel Diameter)			
	ΔR^2^	B (95% CI)	ΔR^2^	B (95% CI)	R^2^	SE
Magnitude (mV)	0.542	1.53 (−0.91–3.98)	0.047	115.50 (−53.95–284.96)	0.589	0.280
MagnitudeD (mV)	0.648	2.45 (−0.074–4.97)	0.092 *	190.34 (31.95–348.74)	0.740	0.263
MagD/Mag ratio	0.485	1.80 (0.89–2.71)	0.169 **	190.34 (31.95–348.74)	0.651	0.090

CCT = central corneal thickness, SE = spherical equivalent. Steps of the regression analysis: ΔR^2^ is the change in R^2^, B (95% CI) is the B coefficient and 95% confidence interval ranges, R^2^ is the total R2 of the model, and SE is the standard error of the estimates of the final model. * *p* < 0.05. ** *p* < 0.01.

**Table 4:** Associations of ganglion cell layer-inner plexiform layer thickness measurements with retinal vessel diameter, controlling for age, signal strength, and central corneal thickness.

	Step 1 (Age, Signal Strength, and CCT)		Step 2 (Vessel Diameter)			
GCL-IPLT Sectors	ΔR^2^	B (95% CI)	ΔR^2^	B (95% CI)	R^2^	SE
Average	0.139	93.4 (38.1–160.8)	0.286 **	4039.4 (1291.2–6787.6)	0.304	5.440
Minimum	0.076	84.8 (19.0–150.6)	0.371 **	4759.9 (1966.6–7553.1)	0.447	5.530
Superior	0.280	136.6 (77.9–195.3)	0.231 **	3792.0 (1139.0–6445.0)	0.510	5.252
Superonasal	0.253	131.4 (67.0–195.9)	0.206 *	3865.8 (859.5–6872.2)	0.459	5.952
Superotemporal	0.272	124.5 (68.8–180.1)	0.110	2466.2 (−346.2–5278.8)	0.382	5.568
Inferior	0.322 *	137.6 (79.1–196.0)	0.164 *	3288.4 (487.4–6075.5)	0.486	5.522
Inferonasal	0.300	134.4 (71.3–197.5)	0.196 *	3810.6 (872.3–6748.9)	0.495	5.817
Inferotemporal	0.210	106.1 (52.1–160.0)	0.088	2053.4 (−733.5–4840.4)	0.298	5.517

CCT = central corneal thickness, GCL-IPLT = ganglion cell layer-inner plexiform layer thickness. Steps of the regression analysis: ΔR^2^ is the change in R^2^, B (95% CI) is the B coefficient and 95% confidence interval ranges, R^2^ is the total R2 of the model, and SE is the standard error of the estimates of the final model. * *p* < 0.05. ** *p* < 0.01.

**Table 6:** Associations of ganglion cell layer-inner plexiform layer thickness measurements with magnitudeD, controlling for age, signal strength, and central corneal thickness.

	Step 1 (Age, Signal Strength, and CCT)		Step 2 (MagnitudeD)			
GCL-IPLT Sectors (µm)	ΔR^2^	B (95% CI)	ΔR^2^	B (95% CI)	R^2^	SE
Average	0.521 **	112.5 (71.5–153.4)	0.130 *	5.9 (1.3–10.6)	0.652	4.237
Minimum	0.445 **	97.5 (51.9–143.2)	0.185 **	7.3 (2.3–12.3)	0.630	4.522
Superior	0.522 **	125.2 (82.4–167.9)	0.179 **	7.3 (2.8–11.8)	0.701	4.103
Superonasal	0.605 **	125.1 (83.2–167.0)	0.062	4.6 (−0.5–9.7)	0.666	4.675
Superotemporal	0.432 **	108.6 (64.6–152.6)	0.173 **	6.8 (1.9–11.7)	0.605	4.452
Inferior	0.801 **	134.9 (106.6–163.2)	0.014	2.1 (−1.6–5.7)	0.815	3.316
Inferonasal	0.668 **	131.4 (92.6–170.3)	0.062 *	4.7 (0.0–9.4)	0.731	4.250
Inferotemporal	0.669 **	105.6 (74.3–136.8)	0.040	3.0 (−0.9–6.9)	0.709	3.550

CCT = central corneal thickness, GCL-IPLT = ganglion cell layer-inner plexiform layer thickness. Steps of the regression analysis: ΔR^2^ is the change in R^2^, B (95% CI) is the B coefficient and 95% confidence interval ranges, R^2^ is the total R2 of the model, and SE is the standard error of the estimates of the final model. * *p* < 0.05. ** *p* < 0.01.

**Table 7:** Associations of ganglion cell layer-inner plexiform layer thickness measurements with magnitudeD/magnitude ratio, controlling for age, signal strength, and central corneal thickness.

	Step 1 (Age, Signal Strength, and CCT)		Step 2 (MagD/Mag Ratio)			
GCL-IPLT Sectors	ΔR^2^	B (95% CI)	ΔR^2^	B (95% CI)	R^2^	SE
Average	0.521 **	112.5 (71.5–153.4)	0.185 **	24.6 (9.7–39.5)	0.706	3.892
Minimum	0.445 **	97.5 (51.9–143.2)	0.298 **	32.4 (17.9–46.8)	0.744	3.762
Superior	0.522 **	125.2 (82.4–167.9)	0.093 *	18.2 (0.4–36.0)	0.615	4.658
Superonasal	0.605 **	125.1 (83.2–167.0)	0.017	8.4 (−10.7–27.4)	0.622	4.980
Superotemporal	0.432 **	108.6 (64.6–152.6)	0.075	15.5 (−3.5–34.6)	0.507	4.973
Inferior	0.801 **	134.9 (106.6–163.2)	0.013	7.1 (−5.6–19.8)	0.814	3.319
Inferonasal	0.668 **	131.4 (92.6–170.3)	0.014	7.7 (−9.9–25.4)	0.682	4.614
Inferotemporal	0.669 **	105.6 (74.3–136.8)	0.048	11.5 (−1.9–24.9)	0.717	3.503

CCT = central corneal thickness, GCL-IPLT = ganglion cell layer-inner plexiform layer thickness. Steps of the regression analysis: ΔR^2^ is the change in R^2^, B (95% CI) is the B coefficient and 95% confidence interval ranges, R^2^ is the total R2 of the model, and SE is the standard error of the estimates of the final model. * *p* < 0.05. ** *p* < 0.01.

## Data Availability

The original contributions presented in this study are included in the article. Further inquiries can be directed to the corresponding author.
